# Interlocking detachable coil embolization for giant tandem bronchial aneurysms: A case report

**DOI:** 10.1097/MD.0000000000028416

**Published:** 2021-12-23

**Authors:** Yingjie Chen, Wei Qin, Ziyang Zhu, Xiaojiang Wang, Wei Yu, Fajiu Li, Chenghong Li

**Affiliations:** a School of Medicine, Jianghan University; b Department of Pulmonary and Critical Care Medicine, Pulmonary Vascular Interventional Centre, Hemoptysis Centre, Affiliated Hospital of Jianghan University; c Institution of Pulmonary Vascular Disease, Jianghan University, China.

**Keywords:** bronchial artery aneurysm, bronchial artery embolization, interlocking detachable coils

## Abstract

**Rationale:**

Bronchial artery aneurysm (BAA) is a rare disease that can be life-threatening if it ruptures. Tandem connections of multiple aneurysms are even rarer and more challenging to manage.

**Patient concerns:**

A 46-year-old woman presented to the hospital with intermittent hemoptysis for a week. A bronchial artery computed tomographic angiography scan revealed 2 BAAs associated with bronchial artery-to-pulmonary artery fistulas in the left lung. Three-dimensional CT reconstruction showed 2 bronchial aneurysms in tandem and 1 aneurysm adjacent to the descending aorta.

**Diagnoses:**

Giant tandem bronchial aneurysms were confirmed using computerized tomographic angiography.

**Interventions:**

Nine interlocking detachable coils and 11 standard pushable coils were introduced into aneurysms for embolization.

**Outcomes:**

There was no episodes of hemoptysis. CT angiography indicated that the coils were closely knit and in their proper position 1 month later; at follow-up, the patient had no adverse effects and no recurrence of hemoptysis.

**Lessons:**

BAA is a rare disease that can be life-threatening if it ruptures. It should be treated aggressively to determine the presence of symptoms.

## 1 Introduction

Bronchial artery aneurysm (BAA) is a rare condition that can be life-threatening if it ruptures.^[1]^ Therefore, it should be treated aggressively, regardless of the presence or absence of symptoms. At present, the etiology of BAA remains unclear. Some scholars have suggested that increased bronchial artery blood flow and focal weakening of the vessel wall are involved in the pathogenesis of BAA.^[2]^ BAA exhibits different clinical manifestations, depending mainly on the primary site, size, and rupture status. The most common clinical symptoms reported in the literature are chest pain and hemoptysis. In asymptomatic patients, BAA is usually detected on chest CT examination. Bronchial artery computed tomography angiography (CTA) can provide important clues and a basis for the diagnosis of BAA. Digital subtraction angiography (DSA) is the gold standard for the diagnosis of BAA. In this report, we present a rare case of multiple BAAs in series with the same bronchial artery and presenting as hemoptysis. This patient presented with massive hemoptysis on admission and was successfully treated with bronchial artery embolization using interlocking detachable coils (IDCs), standard pushable coils, polyvinyl alcohol particles (PVAs), and gelatin sponge (GS) particles.

## 2 Case presentation

**Figure 1:**
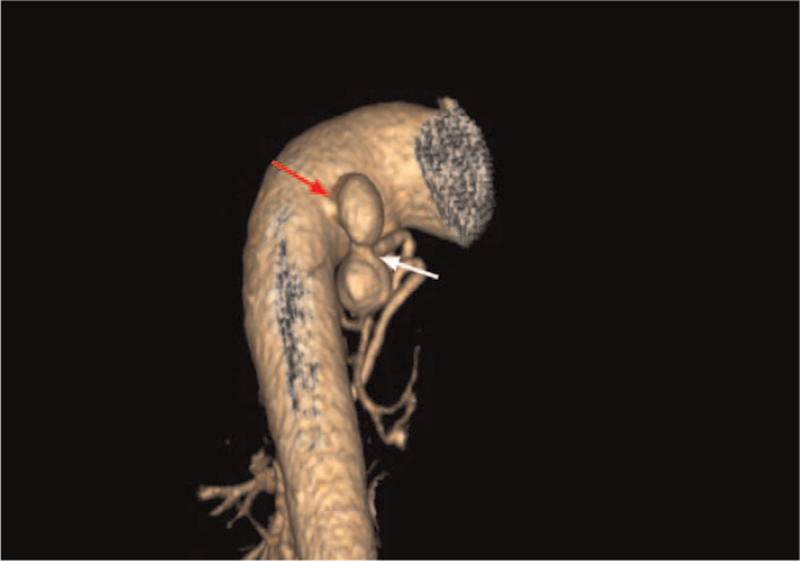
Three-dimensional CT reconstruction of bronchial artery aneurysms. These 2 aneurysms were found in tandem on the same bronchial artery. The first aneurysm (red arrow) originated directly from the aorta and had a narrow neck between itself and the second aneurysm (white arrow).

A 46-year-old woman presented to the hospital with intermittent hemoptysis for a week, resulting in bleeding of up to 500 mL on each occasion. She had a medical history of bronchiectasis that had returned 30 years ago. She had no history of hypertension, diabetes mellitus, or coronary heart disease. On admission, she had a temperature of 36.5°C, heart rate of 78 beats/min, respiratory rate of 22 breaths/min, oxygen saturation of 98% (Fio2 = 29%), and blood pressure of 113/69 mm Hg. There were no remarkable laboratory results. A bronchial artery CTA scan revealed 2 BAAs associated with bronchial artery-to-pulmonary artery fistulas in the left lung. Three-dimensional CT reconstruction showed 2 tandem bronchial aneurysms, one of which was adjacent to the descending aorta (Fig. 1). These 2 bronchial aneurysms had almost the same diameter of approximately 15 mm.

**Figure 2:**
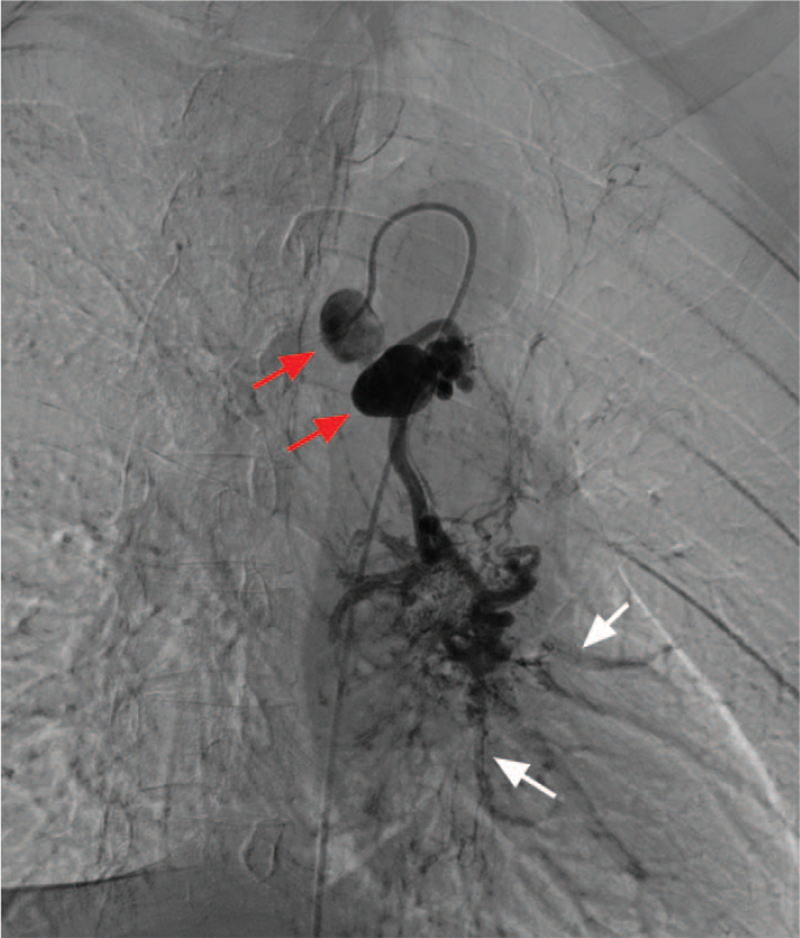
Selective bronchial arteriography showed these 2 aneurysms (red arrow) and the multiple bronchial-artery-to-pulmonary-artery fistulas (white arrow).

**Figure 3:**
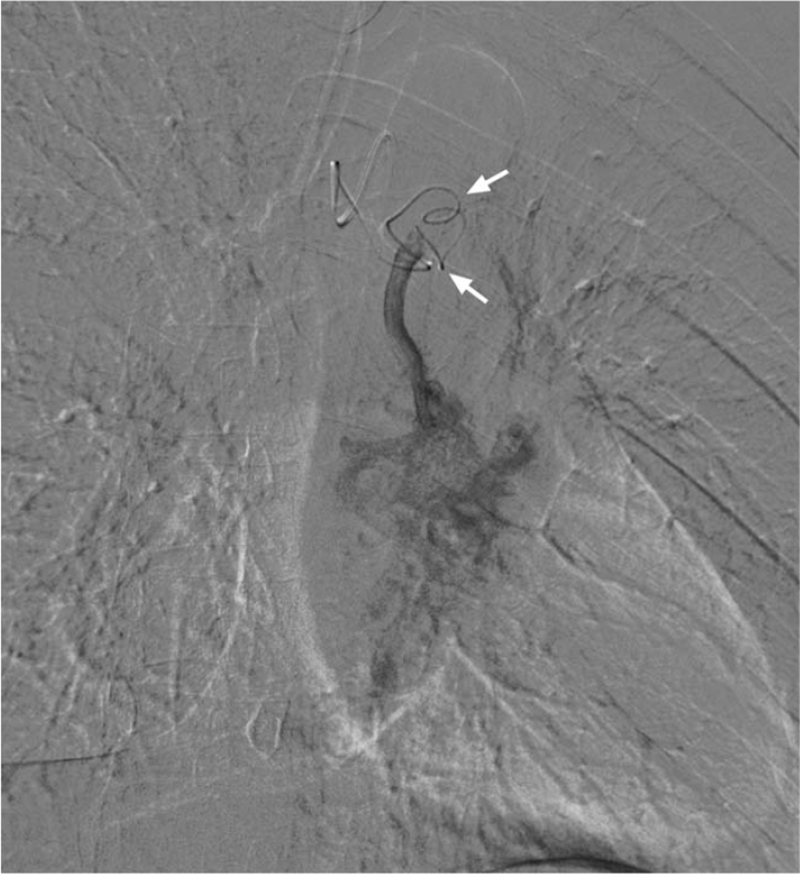
A 1.98F microcatheter coaxially introduced through a 5F MIK catheter, crossing through all aneurysms for distal angiography and granular embolization of the vascular bed.

**Figure 4:**
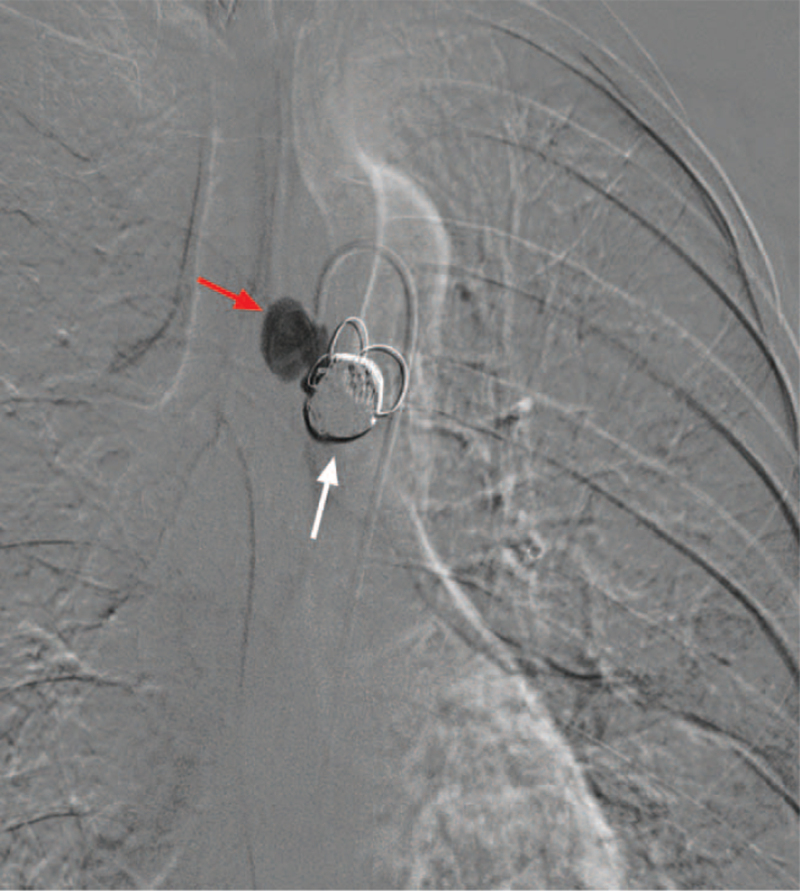
Arteriography after the embolization of the first aneurysm. Aneurysms with (white arrow) and without (red arrow) embolization are shown.

**Figure 5:**
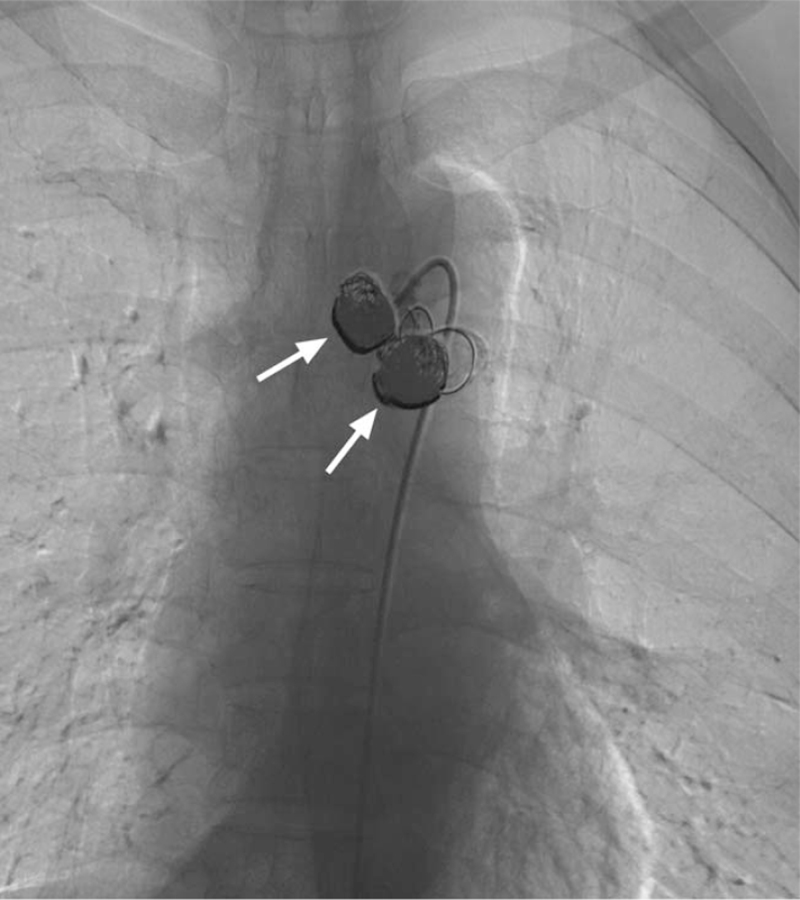
Arteriography after the embolization of vascular bed and aneurysms. Blood vessels are no longer visualized.

**Figure 6:**
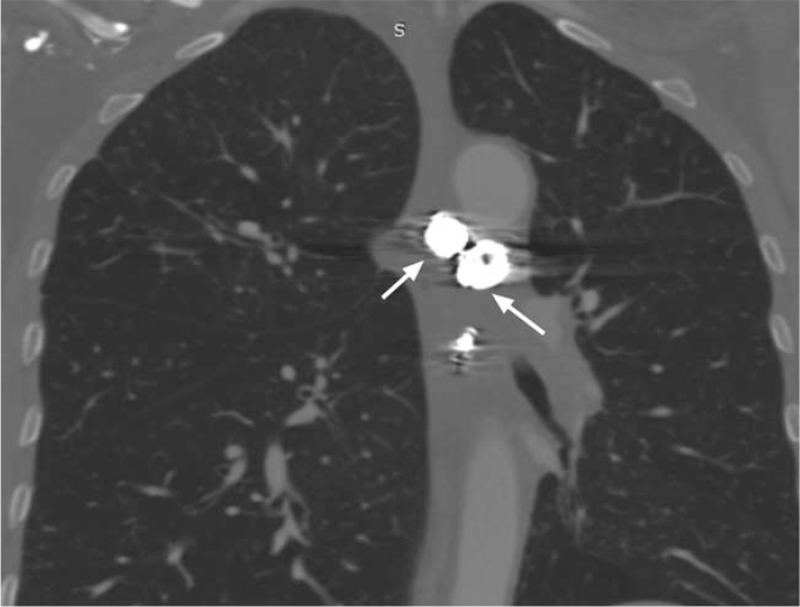
One-month follow-up CT angiography indicates that the coils were closely knit and in their proper position.

After administration of local anesthesia, bronchial arteriography was performed using a 5-F Cobra-3 catheter (Cook Medical) based on CTA images to identify abnormal vessels. Selective bronchial arteriography showed these 2 aneurysms and multiple bronchial-artery-to-pulmonary-artery fistulas (Fig. 2). The vessels were selectively catheterized using a 1.98F microcatheter (Terumo, Tokyo, Japan) coaxially introduced through a 5-F MIK catheter (Cook Medical). For aneurysm embolization, a microcatheter was introduced through all aneurysms for distal angiography, and then the vascular bed was embolized using 300 to 500 μm PVA (Cook Medical) and 560 to 710 μm GS (Hangzhou Alicon Pharmaceutical Co., Ltd., Hangzhou, China) (Fig. 3). Subsequently, 4 IDCs (Boston Scientific) were slowly introduced into the first aneurysm and formed into a nest. Standard pushable coils (10 mm × 14 cm, Cook Medical) were used to reinforce the nest (Fig. 4). These 2 aneurysms were embolized one after another, using a total of 9 IDCs and 11 standard pushable coils. After embolization of the vascular bed and aneurysms, the blood vessels were no longer visible on the angiogram (Fig. 5). After aneurysm embolization was completed, the remaining 2 anomalous vessels were embolized sequentially. One month later, CT angiography indicated that the coils were closely knit and in their proper position (Fig. 6).

## 3 Discussion

Multiple BAAs are rare but can be life-threatening. According to the literature, bronchial aneurysms are present in less than 1% of all patients undergoing selective bronchial arteriography.^[3]^

The exact causes of BAA formation are unknown but can be grouped into 2 types: congenital and acquired.^[4]^ Diseases related to BAA include bronchiectasis, chronic obstructive pulmonary disease, pulmonary vascular inflammation, chronic bronchial and lung infections, tuberculosis, cystic fibrosis, and hereditary hemorrhagic telangiectasia.^[5,6]^ Some patients with BAA had no clear history of the disease. In this case, the aneurysm was caused by chronic inflammation of the bronchial dilatation with long-term irritation of the blood vessels, but the exact mechanism of formation needs to be further investigated.

No typical clinical manifestations of bronchial aneurysms are present, and they are usually detected incidentally during chest CT.^[7]^ BAA may be located in the mediastinum or lungs.^[8]^ Difficulty in swallowing and facial edema due to compression of the superior vena cava may occur when the BAA is large and occurs in the mediastinum; when it ruptures in the mediastinum, chest pain is the most common symptom.^[9]^ In severe cases, it can cause hemorrhagic shock. A small number of aneurysms can break into the esophagus and cause vomiting.^[10]^ In addition, if the aneurysm is located in the lung, massive hemoptysis can occur if it ruptures.^[11]^

Transcatheter vascular embolization for the treatment of aneurysms has been extensively reported in the literature, mainly in the selection of embolic substances.^[2,12–14]^ N-butyl cyanoacrylate (NBCA), PVA pellets, GS pellets, standard pushable coils, and covered endovascular stents were used alone or in combination for embolization of the BAA. NBCA is a liquid embolization material that is primarily indicated for arterial embolization in bronchial aneurysms or dilated tortuous bronchial arteries that are difficult to insert.^[15]^ In a recent retrospective observational single-center cohort study, 58 (100%) patients with pseudoaneurysms were successfully embolized, indicating that NBCA is a definitive and safe embolization agent.^[16]^ However, in the present case, NBCA was not a good choice because the aneurysm was directly attached to the aorta, and the liquid embolic agent could easily flow backward and cause ectopic embolism. Spring coils are primarily indicated in patients with arterial catheters that can successfully reach the BAAs, but conventional coils do not have a controlled systemic device, and there is an inherent risk that they cannot be removed once they are released into the aneurysm or vessel.^[1]^ Unlike conventional spring-loaded coils, IDCs are made of platinum and have a unique interlocking arm structure that can be withdrawn before they are fully released, ensuring precise control of positioning and release. IDCs can be removed when they are not properly sized or inaccurately positioned for release. IDC is widely used for clinical interventions such as aneurysms and arteriovenous fistulas, but the use of embolization for bronchial aneurysms has been less reported.^[12]^

In this case, the BAA on the bronchial artery was embolized with IDCs and general spring coils, while the vascular bed was embolized with a granular embolic agent. The microcatheter was inserted distal to the aneurysm to prevent ectopic embolization due to regurgitation of the granular embolic agent into the aorta and to ensure that the vascular bed of the anomalous vessel was adequately embolized. In addition, the use of IDC can reduce complications and hospitalization costs without the use of anticoagulation therapy after stent implantation. To date, the patient has no adverse effects or recurrence of hemoptysis and is still under long-term follow-up.

## 4 Conclusion

BAA is a rare disease, and early imaging examinations are of great significance for a clear diagnosis. The IDC has the advantages of complete controllability, compact packing, and good transportability. It is an ideal vascular embolization material that can be used in the embolization treatment of complex aneurysms or vascular malformations.

## Acknowledgments

We acknowledge all our colleagues for their selfless contributions in drafting this article. The patient provided informed consent for the publication of this case.

## Author contributions

**Conceptualization:** Yingjie Chen, Wei Qin, Fajiu Li, Chenghong Li.

**Data curation:** Yingjie Chen, Wei Qin, Ziyang Zhu, Xiaojiang Wang, Wei Yu, Fajiu Li.

**Formal analysis:** Yingjie Chen, Wei Qin, Ziyang Zhu, Xiaojiang Wang, Fajiu Li, Chenghong Li.

**Funding acquisition:** Ziyang Zhu.

**Investigation:** Yingjie Chen, Wei Qin, Wei Yu.

**Methodology:** Yingjie Chen, Wei Qin, Xiaojiang Wang.

**Resources:** Ziyang Zhu, Xiaojiang Wang, Wei Yu, Fajiu Li, Chenghong Li.

**Software:** Wei Qin, Ziyang Zhu, Wei Yu.

**Supervision:** Fajiu Li, Chenghong Li.

**Visualization:** Wei Qin, Fajiu Li.

**Writing – original draft:** Yingjie Chen, Wei Qin, Ziyang Zhu, Fajiu Li, Chenghong Li.

**Writing – review & editing:** Fajiu Li, Chenghong Li.
